# Stonefish envenomation of hand with impending compartment syndrome

**DOI:** 10.1186/s12995-016-0112-y

**Published:** 2016-05-10

**Authors:** Terence Khai Wei Tay, Han Zhe Chan, Tunku Sara Tunku Ahmad, Kok Kheng Teh, Tze Hau Low, Nuraliza Ab Wahab

**Affiliations:** Department of Orthopaedic Surgery, Faculty of Medicine, University of Malaya, 50603 Kuala Lumpur, Malaysia

**Keywords:** Stonefish, Synanceia, Bites and stings, Marine toxins, Impending compartment syndrome

## Abstract

**Background:**

Marine stings and envenomation are fairly common in Malaysia. Possible contact to various marine life occurs during diving, fishing and food handling. Even though majority of fish stings are benign, there are several venomous species such as puffer fish, scorpion fish, lionfish, stingray and stonefish that require urgent medical treatment. Stonefish is one of the most venomous fish in the world with potential fatal local and systemic toxicity effects to human.

**Case Presentation:**

We reported a case of stonefish sting complicated with impending compartment syndrome.

**Conclusions:**

Medical staff should be alert about the possibility of this potential emergency in standard management of stonefish stings.

## Background

*Synanceia* spp. is a genus of fish from the family Synanceiidae, the Stonefish, whose members are dangerous and even fatal to human. It is one of the most venomous fish currently known in the world [1]. They are found in the coastal regions of Indo-Pacific oceans as well as off the coast of Florida and in the Caribbean.

Stonefish has potent neurotoxins secreted from glands at the base of their needle-like dorsal fin spines, which stick up when disturbed or threatened. The name, “Stonefish”, derives from its ability to camouflage with a grey and mottled color similar to the color of a stone. They are often covered by a coat of slime to which algae adhere. This excellent camouflage and their habit of partially burying themselves in the sand, makes them difficult to detect and avoid. Many small fishes easily fall prey to its superior camouflage ability. Human too, may not notice them most of the time, and thus inadvertently touch or step on them, triggering a sting. When disturbed, the stonefish injects an amount of venom proportional to the amount of pressure applied to it. They have earned labels such as ‘the master of deceit’ and ‘devil-fish’ due to their unaesthetic appearance and toxic venom. Like other venomous animal venoms, stonefish venom exhibits hemorrhagic, hemolytic, and proteolytic enzymatic activity [2]. The stings will lead to excruciating pain and gross oedema of the affected limb.

In Malaysia and Singapore, stonefish stings are rare. There were only eight cases reported within year 2001 and 2003 [3].

## Case report

Our patient is a thirty-year-old Burmese aquarium cleaner in Aquaria KLCC, who was accidentally stung on his right index finger by a stonefish during a cleaning work. He immediately suffered from intense burning pain over radial aspect of his right index finger. The sharp pain soon spread to his ipsilateral axilla. Rapid-onset gross swelling developed in the affected finger and within two hours, his entire right index finger and radial half of the hand were swollen and erythematous. There was no stonefish spine retained in the wound. Despite receiving regular analgesia in the form of fentanyl and morphine, he still suffered from intense pain over his index finger. Anti-tetanus injection, antihistamine and steroid were administered and the limb elevated in attempt to reduce the swelling. Intravenous antibiotics (amoxicillin/clavulanic acid and metronidazole) were also given.

**Fig. 1:**
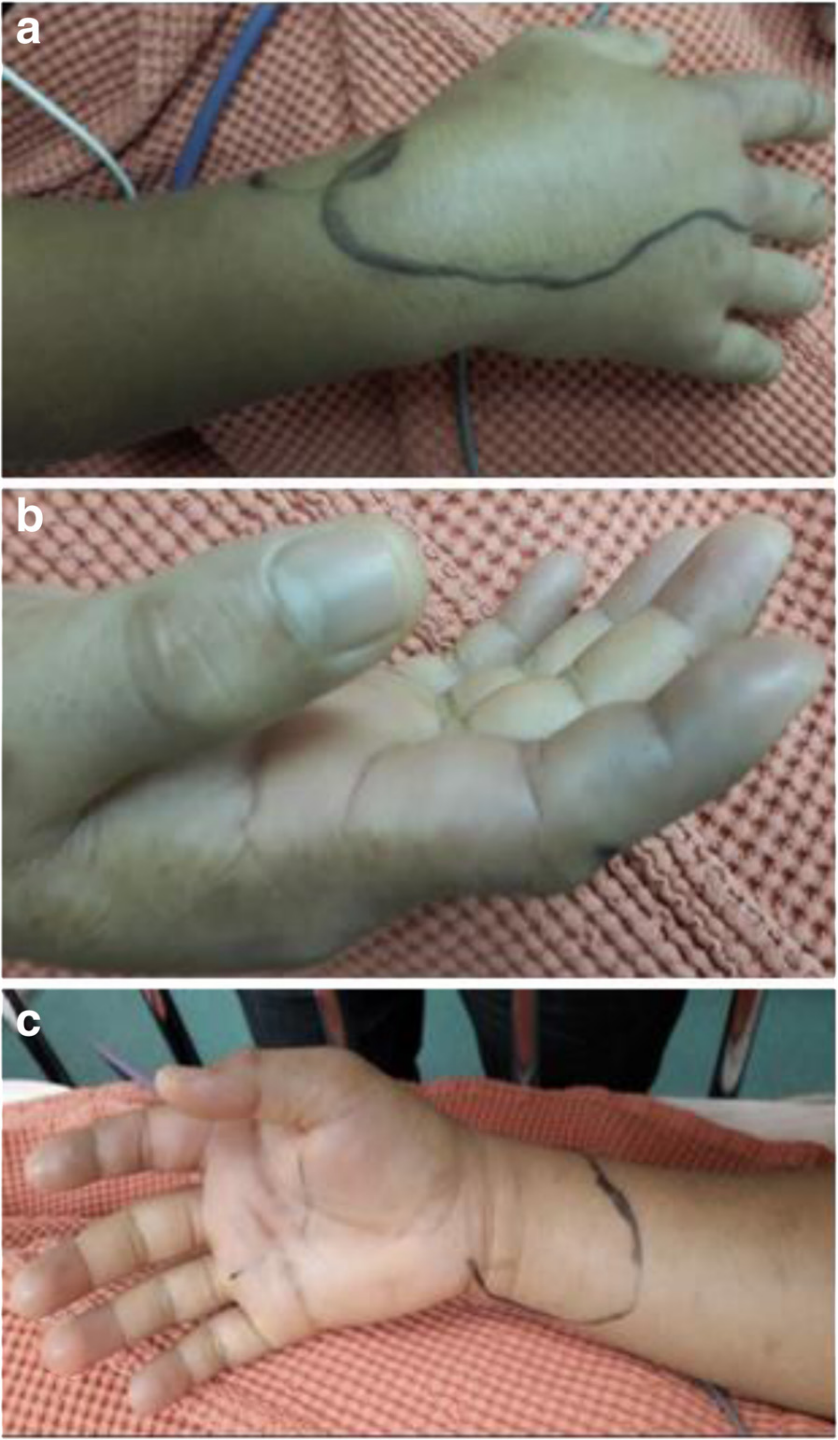
Extensive erythema, gross swelling up to the entire index finger, dorsum, and distal third forearm

Twelve hours later, the pain had progressively worsened again and swelling had spread to involve the dorsum and radial half of his right hand (Fig. 1). Blisters appeared on his right index finger and he had paraesthesia over the median nerve distribution over the affected hand. Sensation reduced over the tip of right index finger and oxygen saturation on pulse oximeter dropped to 89 %, which raised the fear of impending compartment syndrome associated with symptoms of acute carpal tunnel syndrome. Clinically, patient was afebrile with stable vital signs. Laboratory results, including a white blood cell count, haemoglobin level, clotting profile; renal (electrolytes) and liver function tests were all normal. No gas shadow was seen in the right hand plain radiograph. All Gram stains and subsequent cultures were negative. There was no specific anti-venom for stonefish available in National Poison Centre. Patient was treated with warm water immersion and elevation in between immersion. Swelling gradually subsided. Fasciotomy was not required as the patient had responded well with the conservative management. His right hand swelling and circulation improved gradually. There was no local skin necrosis. Patient was discharged well with good recovery from pain and numbness after 4 days of hospital stay. There was no associated neurogenic or vascular sequelae noted during subsequent follow up. He had a full motion in his fingers and wrists joints at six weeks follow up.

## Discussion

### Bio-toxicity

To date, there has been a tremendous effort to identify the specific bioactive venomous properties of stonefish toxin. The lethal effect of these venoms seems to reside in a signature protein unique to individual species within each family. Within the stonefish family, stonustoxin is the lethal protein from *Synanceja horrida*, trachynilysin from *Synanceja trachynis* and verrucotoxin from *Synanceja verrucosa* [4]. However, exact relationships of the toxin are still inconclusive. The most commonly accepted bioactive agents include the enzyme hyaluronidase, stonustoxin, and trachynilysin. Stonefish **hyaluronidase** has potency many times higher than the enzyme from snake venom [5]. Hyaluronidase, through its ability to break down connective tissue, accounts for the significant necrosis associated with stonefish envenomation [6]. It is believed to be responsible for the rapid spread. **Stonustoxin**, the lethal fraction is another bioactive element of the venom reported to be haemolytic and vasorelaxant. It contributes to extensive oedema after envenomation. Stonutoxin is also a potent hypotensive agent, which has myotoxic and neurotoxic activity as well. Marked hypotension appears to be the primary cause of death in animals in vivo studies [7]. **Trachynilysin** is a neurotoxin that depletes neurotransmitter levels at the synapses resulting in hyperstimulatory neuroblockade. As yet, these findings are largely experimental and further investigations are required before definite conclusions can be drawn.

Research shows that the venom is an unstable protein, with a pH of 6.0 and a molecular weight of 150,000. In vitro, it may be denatured by heat (2 min at 50 °C), acid and alkalis (pH > 9,pH < 4), potassium permanganate and Congo red [8]. Its heat labile nature is the basis for the stonefish sting treatment.

### Management of stonefish injury by Hot Water Immersion (HWI)

Generally, standard stonefish envenomation management protocols include initial resuscitation, symptomatic relief with heat immersion therapy, anti-venom administration for systemic or severe local symptoms, and surgical removal of spines or foreign bodies. The evidence for the treatment of puncture-type stings by this method comes from one small experimental study [9] and a total of 99 reports of its effective use in 110 cases from several papers [10, 11]. This evidence has led to recommendation of this treatment method by organizations such as the International Life Saving Federation and the British Marine Life Study Society. The use of HWI is advised in toxicology guidelines such as Toxbase and the BNF and is supported in all five published review articles on marine envenomation. On the other hand, Tang et al [12] have advised caution with heat immersion, because the warmth may create an optimal environment for the development of vibrio necrotising fasciitis, most guidelines and studies still suggest that hot water immersion therapy is an effective treatment.

Two theories have been proposed on how HWI works. Marine venoms consist of multiple proteins and enzymes, and there is evidence that these become deactivated when heated to temperatures above 50 _˚_C [13]. A long-held view is that deactivation of these heat labile proteins by direct heat application leads to inactivation of the venom. They showed that venom lost its lethality more rapidly at temperatures over 43 ˚ C. However, no significant loss of lethality was seen after exposure to temperatures less than 39 ˚C. The theory of deactivation has been questioned by authors who feel that such direct inactivation would require temperatures so high as to result in burns and tissue necrosis in the patient. Despite the concern of burn injury, there is only a single recorded case of significant thermal burn from over 200 cases of the use of HWI [14]. It is an inexpensive, and as there is reasonable evidence that it can relieve pain after a variety of types of fish sting. An alternative theory is that HWI causes modulation of pain receptors in the nervous system leading to a reduction in pain. Established pain hypotheses such as the gate control theory and the diffuse noxious inhibitory control theories have been proposed as possible mechanisms of action for HWI [15].

The most common methods of application are thermal packs, basins with hot water, and hot showers. Application of hot, but not scalding, water (40–42 ˚C) for 30 min or until the pain resolves is the standard advice. American Journal of Public Health concluded that an exposure to water with maximum temperature of 49° Celcius for 8 min can cause burn injuries. Their studies also showed that prolong exposure to water with temperature about 45° Celcius for 2 h or more will cause scalded injuries. Immersion in water heated to the warmest bearable temperature (40–42 °C) is a relatively safe, easily accessible, and effective first-line management for stonefish injuries.

### Stonefish Anti-venom

Most studies support the use of injected anti-venom as a potent tool in the management of stonefish envenomation [16, 17]. Due to its equine origin the anti-venom could theoretically cause serum sickness or precipitate anaphylactic reactions but there is little documented evidence that these adverse effects occur in practice. Indications for the use of stonefish anti-venom include systemic symptoms, severe pain, paralysis or multiple punctures are present. While the intramuscular (IM) route is well established, intravenous administration remains controversial. In general, one ampule (2000U) of Australia Commonwealth Serum Laboratories (CSL) of Melbourne Stonefish Anti-venom is given intramuscularly for puncture wounds from one or two spines. For three to four spine envenomation two vials are administered.

### Compartment syndrome

Current international stonefish management guidelines mainly focused on heat immersion and anti-venom administration. Surgery has been reserved for removal of foreign bodies, especially the fish spines. Stonefish venom is believed to have hemolytic and proteolytic effect to skin and underlying soft tissue including neurovascular bundles. The vicious cycle of vascular permeability and soft tissue oedema can impinge on peripheral nerves and vessels, which can worsen over time, resembling compartment syndrome. In our case, patient presented with impending compartment syndrome that may have required a fasciotomy surgery. Fortunately, his swelling resolved with the elevation and hot water immersion therapy. If the swelling progresses, fasciotomy would have been warranted.

## Conclusions

The stonefish is an environmental hazard to the unwary swimmer, angler, aquarium cleaner, and chefs. Their unparalleled camouflage ability makes them virtually undetectable and unavoidable. Envenomation can lead to unwanted local morbidity, excruciating pain, gross oedema of the affected limb, and severe systemic reactions have been reported. In majority, most of the cases do not result in protracted morbidity and it only requires prompt recognition, early pre-hospital care, and supportive management for symptomatic relief. We figured out that conservative management with short period of hot water immersion at temperature below 45° is generally safe and it helps to prevent unnecessary surgical intervention.

### Consent

Appropriate written informed consent was obtained for publication of this case report. A copy of the written consent is available by the Editor-in-Chief of this journal.
